# Diversity of macrofungi in Dali University, Yunnan Province, China

**DOI:** 10.3897/mycokeys.128.177967

**Published:** 2026-02-10

**Authors:** Song-Ming Tang, Li-Yan Tang, Juan Zhang, Yi-Han Qu, Zheng-Quan Zhang, Hao-Nan Suo, Lin Li, Xi-Jun Su, Han-Bing Song, Hong-Wei Shen, Zong-Long Luo

**Affiliations:** 1 College of Agriculture and Biological Science, Dali University, Dali 671003, China Co-Innovation Center for Cangshan Mountain and Erhai Lake Integrated Protection and Green Development of Yunnan Province, Dali University Dali China; 2 Co-Innovation Center for Cangshan Mountain and Erhai Lake Integrated Protection and Green Development of Yunnan Province, Dali University, Dali 671003, Yunnan, China College of Agriculture and Biological Science, Dali University Dali China; 3 College of Tea (Pu’er), West Yunnan University of Applied Sciences, Pu'er, China College of Tea (Pu’er), West Yunnan University of Applied Sciences Pu'er China

**Keywords:** Morphology, mushrooms, new species, phylogeny, species diversity, taxonomy

## Abstract

Dali University, located in western Yunnan Province, southwest China, is a picturesque institution surrounded by diverse vegetation types and has rich macrofungal resources. However, the macrofungal diversity within the university campus has remained unexplored. Between 2020 and 2024, a comprehensive investigation was conducted to document the macrofungal species present on campus. The study identified a total of 83 macrofungal species across 11 orders and 35 families, based on morphology and molecular sequence data of 485 collections; among them, three are described as new species: *Clavaria lidaensis*, *C. minirubella* and *Marasmius lidaensis*. *Clavaria lidaensis* is characterized by the incrustations’ fragile basidiomata, solitary, rarely scattered to gregarious, caespitose-connate at the base; fertile part subcylindric to fusiform, soft yellow to dark moderate orange, apex rounded, concolorous with fertile part, becoming dark orange with age; sterile part narrow, concolorous, without tomentum or mycelial patch at the base. *Clavaria minirubella* is characterized by the fragile and simple basidiomata, tubular with obtuse apex; subcylindric to fusiform, dark red fertile part. *Marasmius lidaensis* is characterized by the medium-sized basidiomata, dark orange to slightly desaturated yellow pileus, stipe hollow, and abundant floccose on the surface and mycelium grown at the base. To resolve the taxonomic classification and explore the phylogenetic relatedness of the focal species, a combined dataset of ITS and nrLSU sequences was utilized for maximum likelihood and Bayesian phylogenetic inference. These findings not only enrich the understanding of macrofungal diversity in the region but also highlight the potential for further discoveries and conservation efforts.

## Introduction

Macrofungi constitute a highly significant category of fungi due to their diverse applications in food, medicine, and ecology (Wu et al. 2019; Corrales et al. 2022; Dong et al. 2024, 2025; Niu et al. 2025; Song et al. 2025; Tang et al. 2025a; Wang et al. 2025; Xu et al. 2025; Yang et al. 2025; Zhang et al. 2025). These fungi are highly valued for their edible properties, providing a rich source of nutrients and flavor. In medicine, many macrofungi contain bioactive compounds with potential therapeutic applications, including antioxidants, antimicrobial agents, and immune modulators. Ecologically, they play vital roles in nutrient cycling and soil health. However, it is also important to note that certain macrofungi can act as forest pathogens or produce toxic compounds, posing potential risks to both humans and ecosystems (Sun et al. 2024).

By 2007, global estimates suggested that 53,000–110,000 species of macrofungi existed worldwide (Mueller et al. 2007; Arenas et al. 2018). In China, a comprehensive survey reported 9,302 macrofungi species, including 1,789 edible fungi, 798 medicinal fungi, and 561 species serving both purposes (Dai et al. 2021). More recently, a study documented 558 macrofungal species in southeast Xizang province (Cui et al. 2025); this indicates that southwestern China is rich in fungal resources. Additionally, research has been actively conducted on the species diversity of macrofungi across different regions of China. These studies highlight the ecological, economic, and cultural significance of macrofungi, which play crucial roles in nutrient cycling, soil formation, and plant health through mycorrhizal associations. They also underscore the need for conservation efforts to protect these valuable resources from threats such as habitat loss and overharvesting.

According to the Catalogue of Life China 2025 Annual Checklist, a total of 27,996 fungal species have been discovered in China. In 2025 alone, 1,247 new species and infraspecific taxa were published, including 856 species in the Ascomycota and 20 species in the Basidiomycota.

In China, numerous universities have conducted large-scale surveys on fungal species diversity, with Jiangxi Agricultural University reporting 169 species (Zhang et al. 2018), Hanjiang Normal University 96 species (Wang et al. 2017), Hainan University 88 species (Deng and Wu 2014), Jiangxi Meilin University 77 species (Chai et al. 2014), and Northwest A&F University 73 species (Dou et al. 2015). However, reports on macrofungi diversity from universities in Yunnan Province remain relatively scarce (Lu et al. 2023, 2024).

Dali University, located in Yunnan Province, southwestern China, is a beautiful institution with rich vegetation types and abundant macrofungal resources. Although ecologically important, the diversity of macrofungi in this university campus has been poorly characterized in previous studies. From 2020 to 2024, a comprehensive investigation of macrofungi on campus was conducted to fill this knowledge gap.

Through morphological examination and molecular analyses, we identified 83 macrofungal species, including three new species, they are *Clavaria lidaensis*, *C. minirubella* and *Marasmius lidaensis*.

The *Clavaria* Vaill. ex L. is a member of Clavariaceae Chevallier. Species of this genus are characterized by their simple clavarioid or branched basidiomata (Corner 1950, 1970; Petersen 1988; Yan et al. 2022). At present, approximately 40 species are recognized worldwide, and 13 species have been recorded in China (Tai 1979; Olariaga et al. 2015; Chen and Zhang 2019; Yan et al. 2020, 2022, 2023, 2025).

*Marasmius* Fr. was established by Fries (1836); it is characterized by the small to medium-sized basidiomata, white spore prints, inamyloid basidiospores, and hymeniform pileipellis (Retnowati and Desjardin 2022). Recent molecular phylogenetic analyses have demonstrated the polyphyletic nature of *Marasmius* (Wilson and Desjardin 2005), with 17 species of this genus having been originally described from China (Zhang et al. 2023; Chen et al. 2025).

This study not only enriches the understanding of macrofungal diversity in the region but also highlights the importance of further exploration and conservation efforts. A checklist of the identified macrofungi species is provided, along with GenBank numbers for the sequences obtained from the studied samples. Detailed illustrations, descriptions, and phylogenetic analysis results of the three new species are also included. The findings emphasize the need for continued research on macrofungal diversity in similar environments across China and beyond. Such studies are crucial for understanding the ecological roles of macrofungi, their potential economic value, and the threats they face from habitat loss and climate change. Furthermore, documenting macrofungal diversity contributes to the broader understanding of ecosystem complexity and resilience, while providing opportunities for public education and science outreach. By engaging local communities and students in fungal surveys and conservation efforts, researchers can raise awareness of the importance of fungi in sustaining healthy ecosystems and the need for biodiversity conservation.

## Materials and methods

### Morphological studies

Specimens were photographed in the field; all the important collections data were recorded (Rathnayaka et al. 2025), and specimens were separately wrapped in aluminium foil or kept in a plastic collection box and taken to the laboratory of the Fungal Diversity Conservation and Utilization Team (FDCU) in Northwest Yunnan (Dali University). The fresh basidiomes were macro-morphologically described on the same day of collection. Color identification was performed using the Color Hexa website (www. colorhexa. com) to assign codes (Tang et al. 2025b). After thoroughly drying at 50 °C in a food drier, the specimens were stored in sealed plastic bags and deposited in the Herbarium of Cryptogams, Kunming Institute of Botany, Academia Sinica (KUN-HKAS), or deposited in the Herbarium of Dali University. Dried materials were sectioned under a stereo microscope, transferred onto slides, and mounted in a 5% KOH solution. The morphological structures were observed as described by reference. For microscopic characters, anatomical and cytological characteristics such as basidia, basidiospores, and cystidia were observed and photographed using a Nikon ECLIPSE Ni-U microscope at magnifications up to × 1000. For SEM studies, fragments of the lamellae of the dried material were taken, sputter-coated with gold, and analyzed with a TM4000Plusll in Dali University (Hitachi Japan). The notation [x/y/z] specifies that measurements were made on x basidiospores measured from y basidiomata of z collections. At least 50 basidiospores and 20 basidia were measured from one basidiomata. Basidiospores dimensions are given as (a–) b–c (–d). Where “a” and “d” refer to the minimum and maximum values of all measurements, respectively, b–c presents the range of 95% of the measured values; Q is the length/width ratio of basidiospores, Qm is the average Q of all basidiospores, and is given as Qm ± standard deviation; av. represents the average value of all basidiospores.

### DNA extraction, PCR amplification and sequencing

DNA extraction was conducted according to the protocols provided by Genomic DNA, which was extracted from dried specimens using the Ezup Column Fungi Genomic DNA extraction kit (Sangon China) following the manufacturer’s protocol. Primer pairs for PCR were ITS1/ITS4 for the ITS and LR5/LR0R for the LSU locus. ITS and LSU were amplified as described by Tang et al. (2025a). The PCR amplicons were sent to Sangon Biotech (China) for Sanger sequencing. Sequence reads were assembled in SeqMan II (DNA STAR Inc.).

### Sequence alignment and phylogenetic analyses

The newly generated sequences were checked using BioEdit Sequence Alignment Editor version 7.0.4 (Hall 1999) and assembled using SeqMan (DNAstar, Madison, WI, USA). The sequences were then blasted against the GenBank database using the Basic Local Alignment Search Tool (BLAST) to identify the most closely related sequences. Reference sequences were retrieved, minimally adjusted by hand in BioEdit v.7.0.4 first and then aligned using TrimAl (Salvador et al. 2009). Maximum likelihood (ML) analysis was performed separately for each locus, and the concatenated dataset using RAxML-HPC2 v. 8.2.12 (Stamatakis 2014) as implemented on the CIPRES portal, with the GTR+G model for both genes and 1,000 rapid bootstrap (BS) replicates. The GTR+G model was obtained by MrModeltest 2.2. For Bayesian Inference (BI), the best substitution model for each character set was determined with MrModeltest 2.2 on CIPRES, using the Akaike information criterion. Bayesian analysis was performed using MrBayes ver. 3.2.7a as implemented on CIPRES.

### Identification of species

All collected specimens were subjected to molecular data acquisition. The resulting sequences were compared with those available in the NCBI database using BLAST searches. Sequence similarity was first evaluated, and specimens showing a sequence similarity of ≥ 99% were preliminarily regarded as conspecific. Subsequently, morphological comparisons were conducted based on diagnostic characters of closely related taxa. When no stable or significant morphological differences were observed, the specimens were further considered to represent the same species.

## Results

### Phylogenetic analyses

**Figure 1.**
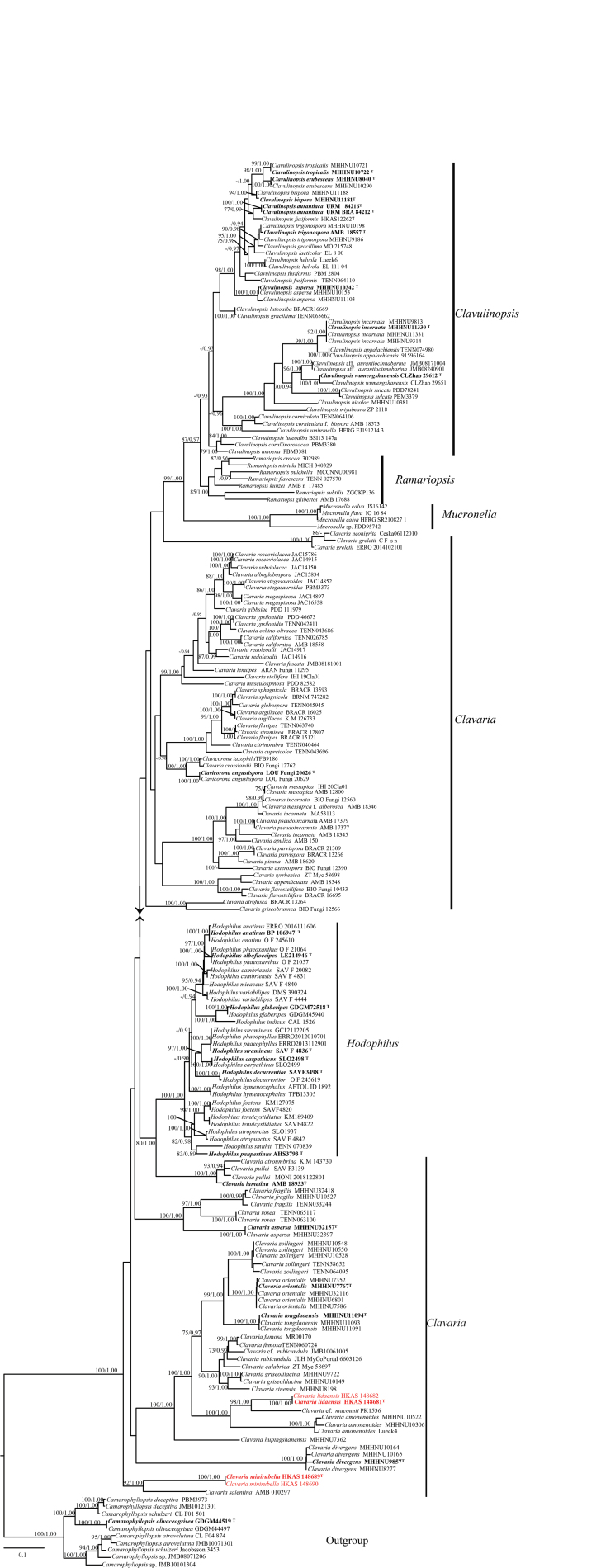
Maximum likelihood phylogeny of ITS and LSU sequence data of *Clavaria*, *Clavulinopsis*, *Hodophilus*, *Mucronella* and *Ramariopsis*, *Camarophyllopsis* was chosen as the outgroup. The new species are highlighted in red; the holotype of each species is in bold.

The phylogeny of Clavariaceae, was constructed using LSU and ITS sequences (Fig. 1); 194 reference specimens and 4 new collected specimens were used for phylogenetic analysis, which comprised 1,707 characters with gaps (LSU = 893, ITS = 814). Single-gene analyses were also performed, and topology and clade stability were compared with those from the combined gene analyses. The final ML optimization likelihood is -38361.761232. The matrix included 1,208 distinct alignment patterns, with 29.77% of characters undetermined or missing. Estimated base frequencies were obtained as follows: A = 0.276304, C = 0.188061, G = 0.250759, T = 0.284876; substitution rates AC = 1.486723, AG = 3.234500, AT = 1.882358, CG = 0.530891, CT = 5.979178, GT = 1.0. ML and BI analyses generated similar topologies, so only the ML tree is presented, along with Maximum likelihood bootstrap (BS ≥ 75%) support values and Bayesian inference (BI) posterior probabilities (PP ≥ 0.90) (Fig. 1). The phylogeny revealed that Clavariaceae was divided into six genera: *Camarophyllopsis* Herink. and *Clavaria* Vaill. ex L., *Clavulinopsis* Overeem, *Hodophilus* R. Heim, *Mucronella* Fr., and *Ramariopsis* (Donk) Corner. Our specimens belong to the *Clavaria* clade and are clearly distinct from other *Clavaria* species.

In the phylogeny of *Marasmius*, which was constructed using ITS and LSU sequences, 65 reference specimens were analyzed, including 38 ITS and 60 LSU. The concatenated ITS and LSU sequence dataset comprised 2,104 characters. The final ML optimization likelihood is -11743.475326. The matrix included 774 distinct alignment patterns, with 33.06% of characters undetermined or missing. Estimated base frequencies were obtained as follows: A = 0.258687, C = 0.198439, G = 0.278994, T = 0.263879; substitution rates AC = 1.195069, AG = 2.359518, AT = 1.442852, CG = 0.692961, CT = 6.013312, GT = 1.0. ML and BI analyses generated similar topologies (Fig. 4), so only the ML tree is presented, along with Maximum likelihood bootstrap (BS ≥ 75%) support values and Bayesian inference (BI) posterior probabilities (PP ≥ 0.90) (Fig. 1). The phylogeny inferred from the ITS and LSU sequences showed that *Marasmius lidaensis* sp. nov. clustered within the *Marasmius* clade. The new species *M. lidaensis* formed a sister lineage to *M. maximus* Hongo (BRNM714571) and *M. brunneospermus* (BRNM 714669).

### Taxonomy

Between 2020 and 2024, a comprehensive investigation was conducted to document the macrofungal species present on campus. The study identified a total of 83 macrofungal species across 11 orders and 35 families (Table 1), based on morphology and molecular sequence data of 485 collections.

**Table 1.** A comprehensive alphabetical inventory (organized by genera) of macrofungi discovered at Dali University, including collection numbers and GenBank accession numbers for sequences derived primarily from the studied specimens.

Taxon	Collection numbers	ITS	Family	Order
*Abortiporus biennis*	20210721-02	PV578017	Podoscyphaceae	Polyporales
*Agaricus abruptibulbus*	x20210718-03	PV578018	Agaricaceae	Agaricales
*Agaricus alboumbonatus*	SJ1186	PV578019	Agaricaceae	Agaricales
*Agaricus brunneovariabilis*	x20210711-08	PV578020	Agaricaceae	Agaricales
*Agaricus campestris*	x20210703-01	PV578021	Agaricaceae	Agaricales
*Agaricus cupreobrunneus*	x20210702-01	PV578022	Agaricaceae	Agaricales
*Agaricus dilutibrunneus*	x20210718-08	PV578023	Agaricaceae	Agaricales
*Agaricus guizhouensis*	Sj1743	PV578024	Agaricaceae	Agaricales
*Agaricus parasubrutilescens*	Sj1988	PV578025	Agaricaceae	Agaricales
*Agaricus xanthodermus*	Sj254	PV578026	Agaricaceae	Agaricales
*Aleuria aurantia*	2020082517	PV578027	Incertae sedis	Pezizales
*Amanita ovalispora*	sj2386	PV578028	Agaricaceae	Agaricales
*Amanita parvipantherina*	x20210727-06	PV578029	Agaricaceae	Agaricales
*Auricularia cornea*	sj267	PV578030	Auriculariaceae	Auriculariales
*Auricularia villosula*	sj268	PV578031	Auriculariaceae	Auriculariales
*Candolleomyces subsingeri*	sj1759	PV578080	Psathyrellaceae	Agaricales
*Clavaria lidaensis*	HKAS 148681	PX353115	Clavariaceae	Agaricales
	HKAS 148682	PX353116		
*Clavaria minirubella*	HKAS 148689	PX353114	Clavariaceae	Agaricales
	HKAS 148690	PX353113		
*Calvatia craniiformis*	sj318	PV578032	Lycoperdaceae	Agaricales
*Candolleomyces candolleanus*	x20210709-01	PV578033	Psathyrellaceae	Agaricales
*Cerrena zonata*	x20210701-01	PV578034	Cerrenaceae	Polyporales
*Cerioporus varius*	x20210726-12	PV578079	Polyporaceae	Polyporales
*Chroogomphus orientirutilus*	sj600	PV578035	Gomphidiaceae	Boletales
*Collybia sordida*	x20210818-04	PV578036	Clitocybaceae	Agaricales
*Collybiopsis subnuda*	f20210902-01	PV578037	Omphalotaceae	Agaricales
*Coltricia weii*	f20210902-10	PV578038	Hymenochaetaceae	Hymenochaetales
*Conocybe apala*	x20210710-03	PV578039	Bolbitiaceae	Agaricales
*Coprinellus xanthothrix*	x20210727-07	PV578040	Psathyrellaceae	Agaricales
*Coprinopsis atramentaria*	x20210708-03	PV578041	Psathyrellaceae	Agaricales
*Coprinus comatus*	x20210711-01	PV578042	Agaricaceae	Agaricales
*Cortinarius lilacinoarmillatus*	f20210901-23	PV578043	Cortinariaceae	Agaricales
*Cortinarius kashmirensis*	f20210901-28	PV578044	Cortinariaceae	Agaricales
*Cyptotrama asprata*	x20210727-12	PV578046	Physalacriaceae	Agaricales
*Echinochaete russiceps*	x20210707-06	PV578047	Polyporaceae	Polyporales
*Entoloma clypeatum*	x20210708-06	PV578048	Entolomataceae	Agaricales
*Flammulina filiformis*	Sj1758	PV578049	Physalacriaceae	Agaricales
*Geastrum triplex*	Sj277	PV578050	Geastraceae	Geastrales
*Gymnopus densilamellatus*	sj356	PV578051	Omphalotaceae	Agaricales
*Gymnopus dryophilus*	20240723-6	PV578052	Omphalotaceae	Agaricales
*Gymnopus dysodes*	x20210717-01	PV578053	Omphalotaceae	Agaricales
*Helvella cremeoinvoluta*	sj647	PV578054	Helvellaceae	Pezizales
*Hygrocybe coccinea*	x20210707-10	PV578055	Hygrophoraceae	Agaricales
*Hygrocybe rubroconica*	x20210831-02	PV578056	Hygrophoraceae	Agaricales
*Hymenochaete rheicolor*	f20210902	PV578057	Hymenochaetaceae	Hymenochaetales
*Hymenopellis raphanipes*	x20210701-03	PV578058	Physalacriaceae	Agaricales
*Hypholoma fasciculare*	Sj2384	PV578059	Strophariaceae	Agaricales
*Infundibulicybe alkaliviolascens*	x20210726-08	PV578060	Clitocybaceae	Agaricales
*Inocybe curvipes*	x20210903-05	PV578061	Inocybaceae	Agaricales
*Kuehneromyces mutabilis*	x20210714-02	PV578063	Strophariaceae	Agaricales
*Laccaria murina*	Sj630	PV578064	Hydnangiaceae	Agaricales
*Lactarius deliciosus*	f20210902-02	PV578065	Russulaceae	Russulales
*Lactarius kesiyae*	x20211024-05	PV578066	Russulaceae	Russulales
*Leotia lubrica*	sj263	PV578067	Leotiaceae	Leotiales
*Mallocybe malenconii*	SJ1170	PV578068	Inocybaceae	Agaricales
*Marasmius lidaensis*	HKAS 148679	PX353111	Marasmiaceae	Agaricales
	HKAS 148680	PX353112		
*Mycena adnexa*	x20210720-03	PV578069	Mycenaceae	Agaricales
*Neofavolus alveolaris*	SJ1768	PV578070	Polyporaceae	Polyporales
*Neofavolus mikawae*	Sj1737	PV578071	Polyporaceae	Polyporales
*Omphalotus flagelliformis*	Sj628	PV578072	Omphalotaceae	Agaricales
*Parasola setulosa*	SJ1990	PV578073	Psathyrellaceae	Agaricales
*Paxillus involutus*	x20210726-21	PV578074	Paxillaceae	Boletales
*Phallus rugulosus*	x20210909-04	PV578075	Phallaceae	Phallales
*Phallus rubrovolvatus*	ZS017	PV578045	Phallaceae	Phallales
*Phylloporus yunnanensis*	f20210902-05	PV578076	Boletaceae	Boletales
*Picipes dictyopus*	x20210718-11	PV578077	Polyporaceae	Polyporales
*Polyporus tuberaster*	x20210718-10	PV578078	Laetiporaceae	Polyporales
*Pseudosperma yunnanense*	SJ2387	PV578062	Inocybaceae	Agaricales
*Trametes coccinea*	x20210726-17	PV578081	Polyporaceae	Polyporales
*Ramaria gracilis*	Sj268	PV578082	Gomphaceae	Gomphales
*Rhizopogon evadens*	Sj1144	PV578083	Rhizopogonaceae	Boletales
*Russula cessans*	Sj339	PV578084	Russulaceae	Russulales
*Russula cremicolor*	f20210902-08	PV578085	Russulaceae	Russulales
*Russula indocatillus*	f20210901-12	PV578086	Russulaceae	Russulales
*Russula pelargonia*	x20210920-01	PV578087	Russulaceae	Russulales
*Russula zhuzuijun*	Sj341	PV578088	Russulaceae	Russulales
*Scleroderma cepa*	Sj335	PV578089	Sclerodermataceae	Boletales
*Suillus huapi*	x20210708-02	PV578090	Boletaceae	Boletales
*Suillus phylopictus*	f20210901-30	PV578091	Boletaceae	Boletales
*Suillus placidus*	Sj1201	PV578092	Boletaceae	Boletales
*Trametes versicolor*	x20210717-06	PV578093	Polyporaceae	Polyporales
*Tricholoma sinoportentosum*	f20210902-11	PV578094	Tricholomataceae	Agaricales
*Tricholoma terreum*	x20210901-05	PV578095	Tricholomataceae	Agaricales
*Xerocomellus fennicus*	x20210714-05	PV578096	Boletaceae	Boletales

The results of the ITS and LSU molecular data comparisons of the three type specimens are shown in Table 2.

**Table 2.** The BLAST result of the three-holotype sequence of the three new species for the closest top five taxa and their corresponding parameters.

Scientific Name	Specimens	Sequence	Voucher	Max score	Total score	Query cover	Per. Ident.	Acc. length	Accession	Reference
*Clavaria lidaensis*	HKAS 148681	ITS	PK1536	521	521	100%	83.03%	658	KP257131	Yan et al. 2025
			P3J09	516	516	99%	82.95%	950	FJ553500	unpublished
			3163	381	381	65%	84.95%	397	MF571435	unpublished
			175487368	364	364	74%	81.82%	658	PP526112	unpublished
			Lueck4	350	350	57%	85.39%	600	KP965768	Azteni et al. 2022
		LSU	AMB018217	1317	1317	99%	94.24%	865	MF972887	unpublished
			PK1536	1291	1291	100%	93.52%	1375	KP257202	Krishnapriya 2023
			KM145803	1258	1258	100%	92.79%	1341	JQ415946	Kautmanová et al. 2012
			Lueck4	1258	1258	100%	92.79%	1026	KP965786	Yan et al. 2025
			AFTOL984	1243	1243	100%	92.45%	1402	AY745693	Kautmanová et al. 2012
*Clavaria minirubella*	HKAS 148689	ITS	6603126	278	278	41%	86.09%	734	MK578690	Krishnapriya and Kumar 2022
			MTS4577	291	291	46%	85.19%	635	KP257114	unpublished
			BMS24-11	291	291	46%	85.19%	622	PQ724464	unpublished
			CF 32637	291	291	46%	85.19%	656	KC759442	Azteni et al. 2022
			KM203191	291	291	46%	85.19%	614	MZ159611	Kautmanová et al. 2012
		LSU	F-684	1240	1240	100%	92.20%	1497	PQ652446	unpublished
			AB0532	1238	1238	100%	92.31%	1324	JQ415935	Kautmanová et al. 2012
			GG AB05-32	1238	1238	100%	92.31%	1048	EF535278	Kautmanová et al. 2012
			BRACR16666	1232	1232	100%	92.19%	943	JQ415961	Kautmanová et al. 2012
			CF32637	1229	1229	100%	92.08%	1326	JQ415945	Kautmanová et al. 2012
*Marasmius lidaensis*	HKAS 148679	ITS	B6	1140	1140	100%	98.32%	678	JX434662	unpublished
			NS16081406	1120	1120	100%	97.57%	688	MN523279	unpublished
			BRNM714571	1105	1105	100%	97.26%	877	FJ904977	Antonín et al. 2010
			KG224	1105	1105	100%	97.26%	850	FJ904974	Antonín et al. 2010
			BRNM714570	1098	1098	100%	97.10%	840	FJ904976	Antonín et al. 2010
		LSU	BRNM714571	1633	1633	100%	99.78%	1381	FJ904959	Antonín et al. 2010
			BRNM714569	1628	1628	100%	99.66%	1410	FJ904949	Antonín et al. 2010
			BRNM714570	1628	1628	100%	99.66%	1420	FJ904958	Antonín et al. 2010
			G1352	1609	1609	99%	99.55%	1282	MK278345	unpublished
			GAL 1672	1598	1598	100%	99.10%	890	NG_064537	unpublished

#### Clavaria lidaensis

Taxon classificationFungiAgaricalesClavariaceae

S.M. Tang & Z.L. Luo sp. nov.

43245800-F955-5D2A-A659-826413B25EDF

Fungal Names: FN 572831

**Figure 2.**
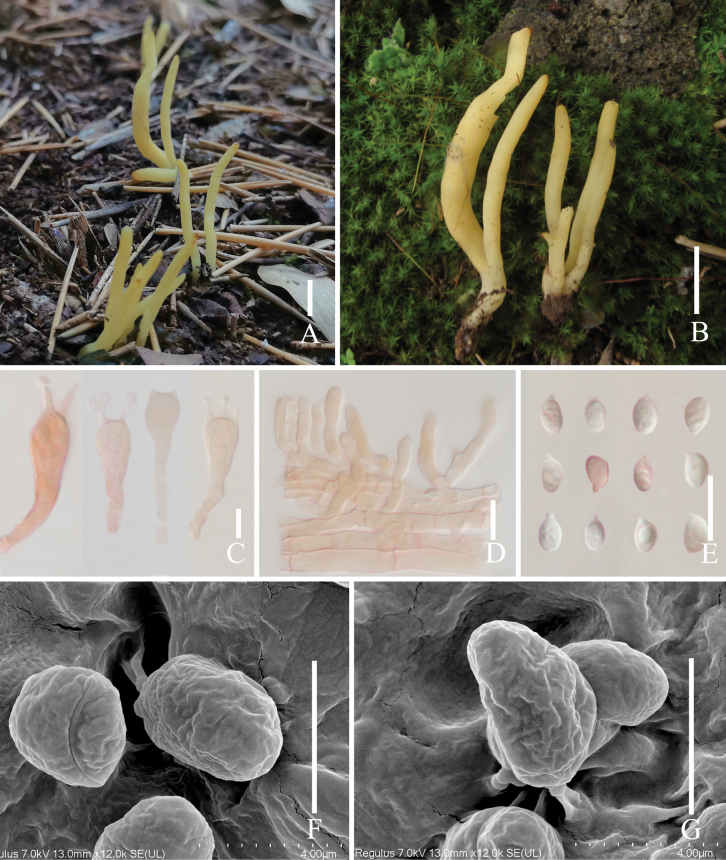
*Clavaria lidaensis*. **A, B**. Basidiomata; **C**. Basidia; **D**. Basidioles in the basidiomata top; **E**. Basidiospores on the optical microscope; **F, G**. Basidiospores on the SEM. Scale bars: 1 cm (**A, B**); 10 μm (**C–E**); 5 μm (**F, G**).

Fig. 2 Chinese Name: 大理大学珊瑚菌(Da Li Da Xue Shan Hu Jun)

##### Etymology.

The epithet “*lidaensis*” refers to the type locality, Dali University (abbreviated as “lida”), where the holotype of this species was collected.

##### Holotype.

China • Yunnan Province, Dali Autonomous Prefecture, Dali University, elev. 2,105 m, September 13, 2020, Jun He, Sj615 (HKAS 148681).

##### Description.

***Basidiomata*** incrustations fragile, simple, 10–30 mm tall, 1–4 mm wide, mostly solitary, rarely scattered to gregarious, mostly caespitose-connate at the base; mostly tubular with acute apex, smooth or irregularly ridged. ***Fertile part*** subcylindric to fusiform, soft yellow (#deca86) to dark moderate orange (#af9755), apex rounded, concolorous with fertile part, becoming dark orange (#824725) with age. ***Sterile part*** narrow, concolorous, without tomentum or mycelial patch at the base. ***Context*** fragile, hymenium concolorous. Taste and odor were not recorded.

***Basidiospores*** (3.5–) 4.3–6.8 (–7.1) × (3.1–) 3.3–4.5 (–4.9) µm, av. 6.0 ± 0.6 × 3.8 ± 0.4 µm, thin-walled, hyaline, smooth, broadly ellipsoid to ellipsoid. ***Basidia*** 30–51 × 7–8 µm, av. 38 ± 7.5 × 7.4 ± 0.5 µm, thin-walled, hyaline, clavate to subcylindrical, rarely spheropedunculate, with a clamp connection, mostly 4–spores, rarely 2–spores, sterigmata 5–7 µm long. ***Basidioles in the basidiomata middle***, 21–36 × 2–7 µm, av. 31.6 ± 6.4 × 4.5 ± 1.9 µm, clavate to subcylindrical. ***Basidioles in the basidiomata top***, 10–15 × 1–5 µm, av. 12.6 ± 1.4 × 2.5 ± 0.9 µm, flexuose, clavate or subcylindrical. ***Subhymenium*** clearly delimited from the context, composed of densely interwoven hyphae. ***Hyphae near subhymenium*** 3.4–7.6 µm wide, cylindrical to inﬂated, thin-walled, hyaline, parallel, without clamp connections. ***Hyphae distant from subhymenium*** 12.1–21.2 (–30.3) µm wide, cylindrical to inﬂated, thin-walled, hyaline, parallel, without clamp connections.

##### Habitat and distribution.

Solitary in mixed forest dominated by *Pinus* sp. and various broadleaved trees; only known from Yunnan Province of Dali Autonomous Prefecture.

##### GenBank accession numbers.

ITS: HKAS 148681, PX353115; HKAS 148682, PX353116; LSU: HKAS 148681, PV802692; HKAS 148682, PV802693.

##### Additional materials examined.

China • Yunnan Province, Dali Autonomous Prefecture, Dali University, elev. 2,108 m, September 13, 2020, Jun He, Sj617 (HKAS 148682).

##### Notes.

*Clavaria lidaensis* and *C. amoenoides* Corner, K.S. Thind & Anand are similar by yellow basidiomata. However, *C. amoenoides* has a larger basidiomata (10–30 vs 50–100 mm tall), and longer basidia ( 30–51 vs 45–60 µm long), and basidiospores 4.3–6.8 × 3.3–4.5 vs 6–9.5 × 3–4.5 µm (Roberts 2008); the ITS base differences between *C. lidaensis* (sj615, holotype) and *C. amoenoides* (MHHNU10551) is 15.3% (85/555 bp).

Phylogenetically, *C. lidaensis* is related to *C. macounii* Peck; however, *C. macounii*, originally distributed from Canada, has a greenish-yellow or pale-cinereous basidiomata, and smaller basidiomata (1.8–3.5 µm; Peck 1894). The ITS base difference between *C. lidaensis* (sj615, holotype) and *C. macounii* (PK1536) is 10.5% (85/810).

#### Clavaria minirubella

Taxon classificationFungiAgaricalesClavariaceae

S.M. Tang & Z.L. Luo sp. nov.

4C40C5BB-C938-515C-BF2E-6D8EA23C4332

Fungal Names: FN 572832

**Figure 3.**
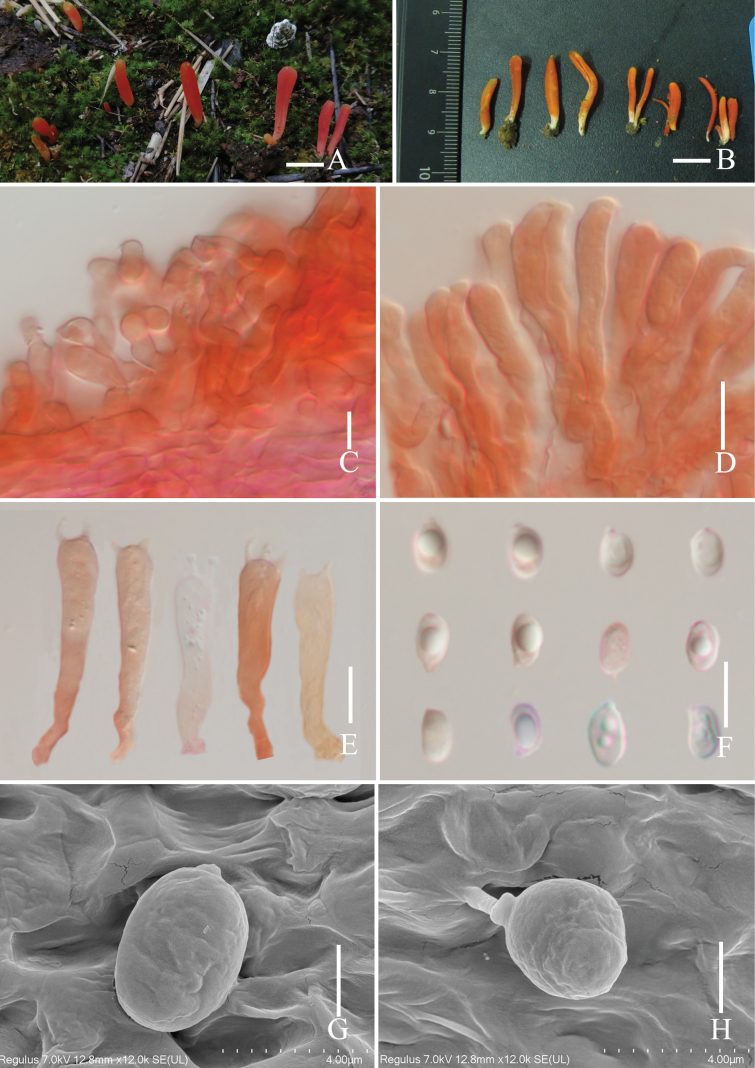
*Clavaria minirubella*. **A, B**. Basidiomata; **C**. Basidioles in the basidiomata top; **D**. Basidioles in the basidiomata middle; **E**. Basidia; **F**. Basidiospores on the optical microscope; **G, H**. Basidiospores on the SEM. Scale bars: 1 cm (**A, B**); 10 μm (**C–E**); 2 μm (**F, G**).

Fig. 3 Chinese Name: 小红珊瑚菌(Xiao Hong Shan Hu Jun)

##### Etymology.

The species epithet “minirubella” refers to the small and red basidiomata.

##### Holotype.

China • Yunnan Province, Dali Autonomous Prefecture, Dali University, elev. 2,005 m, September 13, 2020, Jun He, Sj619 (HKAS 148689).

##### Description.

***Basidiomata*** fragile, simple, 10–20 mm tall, 1–3 mm wide, mostly solitary, rarely scattered to gregarious; mostly tubular with obtuse apex, smooth. ***Fertile part*** subcylindric to fusiform, dark red (#99302d), apex rounded, concolorous with fertile part. ***Sterile part*** narrow, slightly dark moderate red (#a95a51), without tomentum or mycelial patch at the base. ***Context*** fragile, hymenium concolorous. Taste and odor were not recorded.

***Basidiospores*** (4.1–) 5.1–6.8 (–7.1) × (2.7–) 2.9–3.6 (–3.9) µm, av. 5.4 ± 0.4 × 3.3 ± 0.2 µm, thin-walled, hyaline, smooth, broadly ellipsoid to ellipsoid. ***Basidia*** 31–41 × 6–7 µm, av. 35 ± 4.8 × 6.5 ± 0.5 µm, thin-walled, hyaline, clavate to subcylindrical, with a clamp connection, 4–spores, sterigmata 1–3 µm long. ***Basidioles in the basidiomata middle***, 22–33 × 3–6 µm, av. 28.3 ± 3.9 × 4.2 ± 0.7 µm, clavate to subcylindrical. ***Basidioles in the basidiomata top***, 10–20 × 1–4 µm, av. 15.3 ± 1.2 × 2.8 ± 0.5 µm, mostly flexuose, rarely clavate. ***Subhymenium*** clearly delimited from the context, composed of densely interwoven hyphae. ***Hyphae near subhymenium*** 2.8–7.6 µm wide, cylindrical to inﬂated, thin-walled, hyaline, parallel, without clamp connections. ***Hyphae distant from subhymenium*** 7.2–12.1 µm wide, cylindrical to inﬂated, thin-walled, hyaline, parallel, without clamp connections.

##### Habitat and distribution.

Solitary in mixed forest dominated by *Pinus* sp. and various broadleaved trees; only known from Yunnan Province of Dali Autonomous Prefecture.

##### GenBank accession numbers.

ITS: HKAS 148689, PX353114; HKAS 148690, PX353113; LSU: HKAS 148689, PV802694; HKAS 148690, PV802695.

##### Additional materials examined.

China • Yunnan Province, Dali Autonomous Prefecture, Dali University, elev. 2,005 m, September 13, 2020, Jun He, Sj620 (HKAS 148690).

##### Notes.

Our multi-locus phylogenetic analysis indicates that *C. minirubella* is related to *C. salentina* Agnello & Baglivo. However, *C. salentina* is originally distributed from Italy, and the ITS base difference between the specimen *C. salentina* (AMB_010297) and *C. minirubella* (sj619, holotype) is 12.0% (98/814).

**Figure 4.**
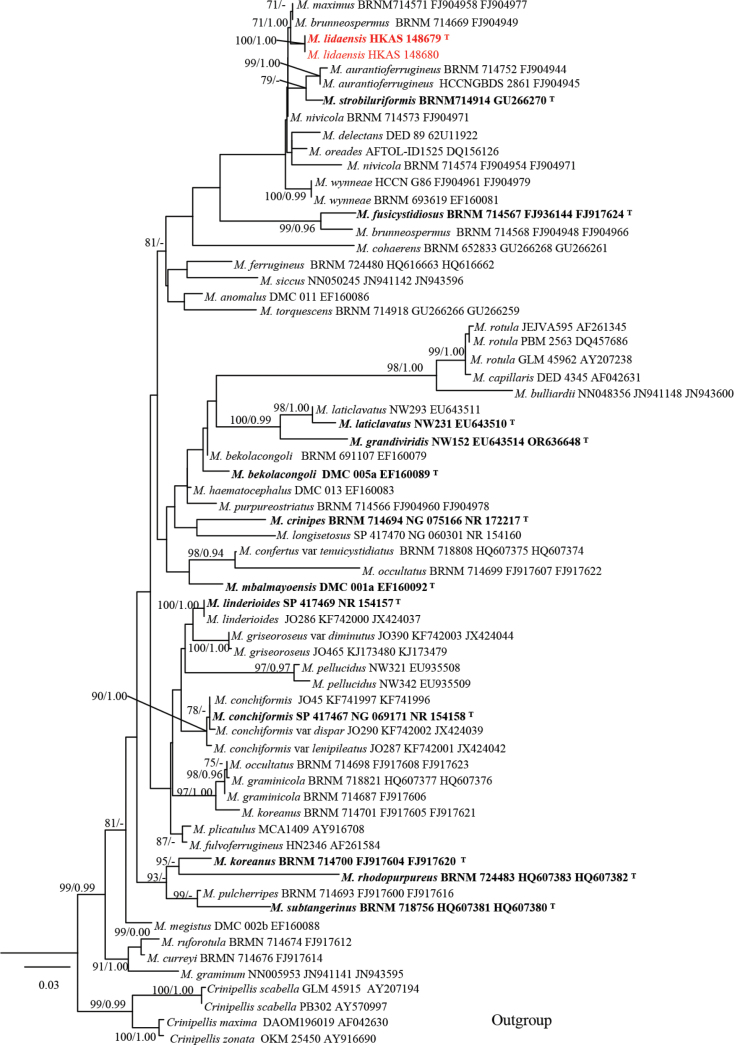
Maximum likelihood phylogeny of ITS1-5.8S-ITS2 and LSU sequence data of *Marasmius*, *Cirnioellis scabella*, *C. maxima* and *C. zonata* were chosen as the outgroup. ML bootstrap (≥ 70%) and posterior probabilities (≥ 0.90) are indicated above branches or in front of the branch leading to each node. The new species are highlighted in red; the holotype of each species is in bold.

Morphologically, *Clavaria minirubella* can be easily confused with *Clavulinopsis sulcata* Overeem because of its strong orange basidiomata (van Overeem 1923). However, the ITS base difference between the specimen *C. minirubella* (sj619, holotype) and *Clavulinopsis sulcata* (PDD_111965) is 31.8% (205/644), thus they are considered to be separate genera.

#### Marasmius lidaensis

Taxon classificationFungiAgaricalesMarasmiaceae

S.M. Tang & Z.L. Luo sp. nov

418F2522-99DE-5ABF-9973-4BF31EBB1587

Fungal Names: FN 572833

**Figure 5.**
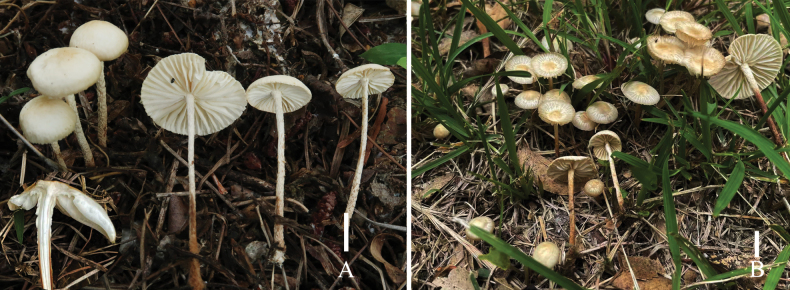
Basidiomata of *Marasmius lidaensis*. **A**. Basidiomata grow in the forest; **B**. Basidiomata grow on the grass. Scale bars: 1 cm.

**Figure 6.**
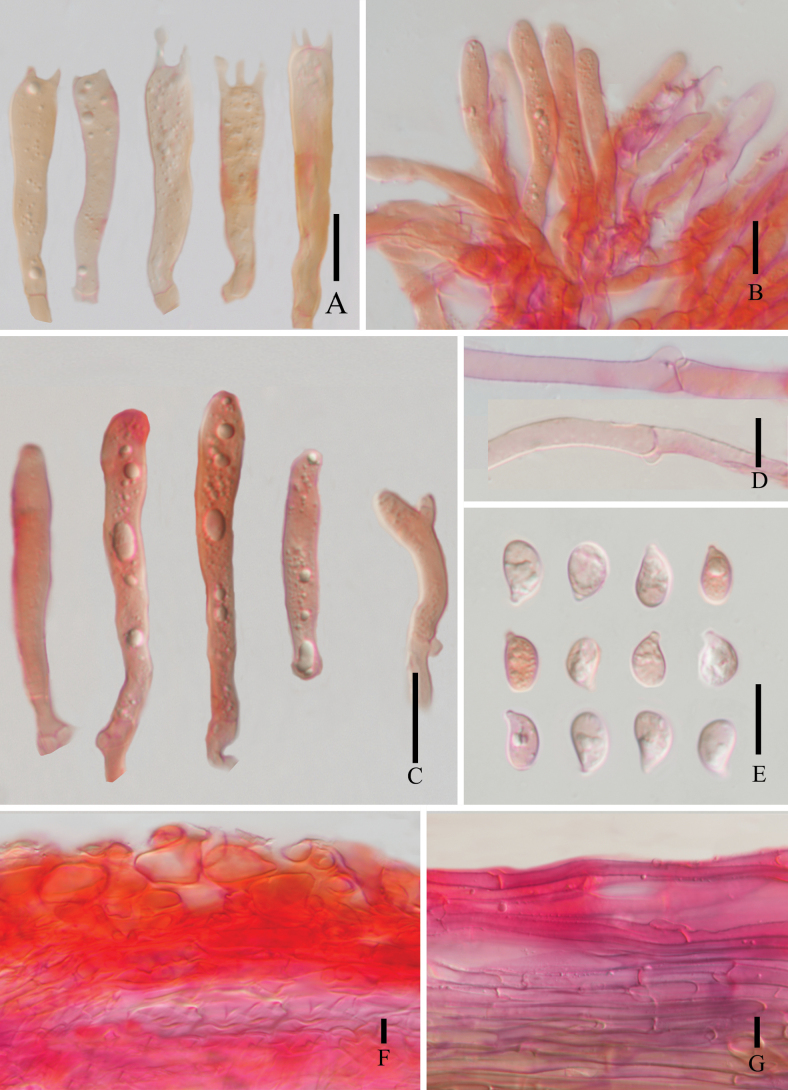
*Marasmius lidaensis* (HKAS 148679, holotype). **A**. Basidia; **B**. Pleurocystidia; **C**. Cheilocystidia; **D**. Clamp connection; **E**. Basidiospores; **F**. Pileipellis; **G**. Stipitipellis. Scale bars: 10 μm.

Figs 5, 6 Chinese Name: 大理大学小皮伞(Da Li Da Xue Xiao Pi San)

##### Etymology.

The epithet “*lidaensis*” refers to the type locality, Dali University (abbreviated as “lida”), where the holotype of this species was collected.

##### Holotype.

China • Yunnan Province, Dali Autonomous Prefecture, Dali University, elev. 2,005 m, September 1, 2020, Jun He, sj523 (HKAS 148679).

##### Description.

***Basidiomata*** medium-sized. ***Pileus*** 20–40 mm in diam., hemispherical when young, becoming convexo-applanate to plano-concave or concave with age, dark orange (#b07530) at center, becoming slightly desaturated yellow (#cabc88) with margin, umbonate of center; aspects exceeding lamellae of margin; distinct, straight, and smooth on the surface; context 1–2 mm thin, tough. ***Lamellae*** 2–4 mm in diam., arcuate when young, ventricose with age, white (#ffffff); attachment emarginate; lamella edge even or entire. ***Stipe*** 30–60 × 1–3 mm, cylindrical, white (#ffffff) on the upwards, becoming dark orange (#724620) with downward, hollow; context soft and tough surface, white (#ffffff) to pale cream; abundant floccose on the surface and mycelium grown at the base. Taste not distinctive.

***Basidiospores*** (5.2–) 5.6–10.2 (–11.5) × (4.2–) 4.5–6.3 (–5.8) μm, av. 8.6 ± 1.03 × 5.5 ± 0.48, Q = 1.1–1.7 μm, ellipsoid, smooth, hyaline, thin-walled. ***Basidia*** 35–42 × 5–7 μm, av. 38.7 ± 4.5 × 5.8 ± 1.32 μm, clavate, with a clamp connection, 4–spored, sterigmata 1–3 μm. ***Basidioles*** 25–52 × 4–6 μm, av. 31 ± 5.2 × 5.2 ± 1.42 μm, scattered, cylindrical, clavate. ***Cheilocystidia*** 24–47 × 4–7 μm, av. 30.5 ± 4.6 × 6.1 ± 0.8 μm, mostly clavate, flexuose, rarely branched, thin-walled. ***Pleurocystidia*** 21–41 × 4–7 μm, av. 31.3 ± 4.1 × 5.7 ± 0.7 μm, abundant, clavate, flexuose, thin-walled. ***Lamellae trama hyphae*** cylindrical or swelling, 4–8 μm in diam., av. 5.9 ± 1.6 μm, thin-walled, with a clamp connection, hyaline in 5% KOH solution. ***Stipitipellis*** a cutis of cylindrical, parallel, thin walled, hyaline in 5% KOH solution, 19–90 × 3–5 μm, av. 50.9 ± 23.7 × 4.0 ± 0.6 μm, present connection camp. ***Pileipellis*** a hymeniderm composed of cells, swelling, 7–17 × 4–11 μm, av. 14.8 ± 3.6 × 8.8 ± 2.0 μm, parallel, hyaline in 5% KOH solution, with a clamp connection.

##### Habitat and distribution.

Solitary in mixed forest dominated by *Pinus* sp. and various broadleaved trees, sometimes basidiomata occur on grass. Only known from the Yunnan Province of Dali Autonomous Prefecture.

##### GenBank accession numbers.

ITS: HKAS 148679, PX353111; HKAS 148680, PX353112; LSU: HKAS 148679, PV802690; HKAS 148680, PV802691.

##### Additional materials examined.

China • Yunnan Province, Dali Autonomous Prefecture, Dali University, elev. 2,007 m, July 8, 2021, Jun He, sj525 (HKAS 148680); June 6, 2024, Song-Ming Tang, 2024060601.

##### Notes.

*Marasmius lidaensis* is distinguished from other *Marasmius* species by its clearly striated pileus surface, dark orange at the center, becoming slightly desaturated yellow with a margin; the stipe is white on the upper side, becoming dark orange on the lower side, with a soft and rigid context surface, and is abundant with floccose hairs on the surface.

According to our multi-locus phylogenetic analyses, *M. lidaensis* was clustered together with *M. maximus* Hongo (BRNM714571) and *M. brunneospermus* Har. Takah. (BRNM 714669). However, *M. maximus* has an ochre-brown to pale brownish to pale ochraceous pileus, and slightly broadened at stipe base, pleurocystidia absent, and irregular lobate cheilocystidia (Hongo 1962); and the ITS base difference between the specimen *M. maximus* (BRNM 714571) and *M. lidaensis* (HKAS 148679, holotype) is 1.3% (10/778). *Marasmius brunneospermus* has a brown to brownish orange pileus, and smaller basidia (35–42 × 5–7 μm vs. 23–27 × 3.3–4.6 μm) (Takahashi 1999); and the ITS base difference between the specimen *M. brunneospermus* (BRNM 714571) and *M. lidaensis* (HKAS 148679, holotype) is 8.7% (68/778).

## Discussion

In this study, we conducted a comprehensive investigation of the diversity of macrofungi on the Dali University campus and in surrounding habitats in Yunnan Province, China. Through a combination of detailed morphological examination and molecular phylogenetic analysis, we identified 83 macrofungi species. These species were distributed across 11 orders and 35 families, reflecting the rich fungal diversity in this region. The majority of the recorded species were widely distributed taxa, further confirming the ecological stability and suitability of the study area for fungal growth.

Remarkably, during our investigation, we discovered and described three species, *Clavaria lidaensis*, *C. minirubella*, and *Marasmius lidaensis*. So far, 22 species of the genus *Clavaria* have been reported in China (Table 3). These species are distributed across ten provinces, namely Anhui, Guizhou, Hubei, Hunan, Jiangxi, Jilin, Shanxi, Xizang, Yunnan and Zhejiang (Yan et al. 2020, 2022, 2025), indicating a wide distribution of the genus in China.

**Table 3.** Selected key morphological characteristics of *Clavaria* species from China.

Taxa	Basidiomata branched or unbranched	Basidiospores size	Basidia size	Basidiomata color	Reference
*C. acuta*	Unbranched	7.0–8.5 × 5.0–7.0 μm	42–55 × 7–10 μm	Pale orange to yellow	Persoon 1822
*C. amoenoides*	Unbranched	4.0–6.0 × 2.5–4.0 μm	35–50 × 5–8 μm	Yellow to very pale orange-yellow	Yan et al. 2022
*C. argillacea*	Mostly Unbranched, rarely branched	9.0–11.0 × 4.0–5.0 μm	51–75 × 6–8 μm	Bright yellow to pale yellow	Olariaga et al. 2015
*C. aspersa*	Unbranched	4.0–5.0 × 2.5–4.0 μm	35–50 × 4–8 μm	White	Yan et al. 2022
*C. asterospora*	Unbranched	5.0–6.0 × 3.8–4.2 μm	27–40 × 4–6 μm	White	Patouillard 1886
*C. divergens*	Branched	4.2–5.0 × 2.7–3.8 μm	48–65 × 6–9 μm	White, becoming yellowish or tawny with age	Yan et al. 2025
*C. falcata*	Unbranched	7.5–9.0 × 5.5–7.0 μm	33–40 × 5–9 μm	White when young, becoming white yellow with age	Persoon 1794
*C. flavipes*	Unbranched	6.0–8.5 × 4.0–6.0 μm	41–60 × 7–8 μm	Pale yellow to light orange	Persoon 1794
*C. fragilis*	Unbranched	4.0–6.0 × 3.0–5.0 μm	26–40 × 5–8 μm	White when young, becoming white yellow or yellow with age	Holmskjold 1790
*C. fumosa*	Unbranched	4.4–5.5 × 3.4–5.0 μm	32–45 × 5–8 μm	Pale yellowish-brown, light gray, or smoky gray.	Persoon 1795
*C. griseolilacina*	Branched	4.0–7.0 × 3.0–5.0 μm	42–57 × 8–10 μm	Pale purple to grayish purple	Yan et al. 2020
*C. gibbsiae*	Unbranched	7.0–10.0 × 6.0–8.0 µm	35–50 × 6–9 µm	Whitish when young, becoming cream, yellowish brown	Yan et al. 2025
*C. hupingshanensis*	Branched	4.0–6.0 × 3.5–5.0 μm	36–50 × 5–8 μm	Rose-white to seashell-pink	Yan et al. 2022
***C. lidaensis***	**Unbranched**	**4.3–6.8 × 3.3–4.5 µm**	**30–51 × 7–8 µm**	**Soft yellow to dark moderate orange**	**This study**
*C. macounii*	Unbranched	6.0–7.0 × 3.0–4.0 µm	30–60 × 5–6 µm	Cream, pale orange-yellow, barium yellow	Peck 1894
***C. minirubella***	**Unbranched**	**5.1–6.8 × 2.9–3.6 µm**	**31–41 × 6–7 µm**	**Dark red**	**This study**
*C. orientalis*	Branched	5.0–6.0 × 4.0–5.0 μm	34–48 × 5–8 μm	Deep amethyst, becoming yellowish or tawny with age	Yan et al. 2025
*C. rubicundula*	Unbranched	6.0–7.0 × 3.5–4.2 μm	32–45 × 6–9 μm	Wine-colored, light yellow, or dark pink	Leathers 1956
*C. rosea*	Unbranched	6.0–8.0 × 3.0–4.0 µm	40–45 × 8 µm	Rose pink, with a white or pallid stem	Yan et al. 2025
*C. sinensis*	Branched	5.0–6.0 × 3.5–4.5 μm	30–40 × 6–8 μm	Pale purple to pinkish-purple	Yan et al. 2020
*C. tongdaoensis*	Branched	3.5–5.0 × 3.0–4.2 μm	26–43 × 6–8 μm	Pale purple to pale purplish pink, becoming yellowish or tawny with age.	Yan et al. 2025
*C. zollingeri*	Branched	5.0–6.0 × 4.0–5.0 μm	34–48 × 5–8 μm	Violet, lilac, or purplish-brown	Léveillé 1846

China exhibits high species richness of the genus *Marasmius*, with more than 140 species recorded to date (Song et al. 2009), which reflects the country’s abundant *Marasmius* germplasm resources. In this study, we systematically collated and comparatively analyzed species morphologically similar to *Marasmius lidaensis* (Table 4), and ultimately confirmed that this fungus represents a distinct species.

**Table 4.** Selected key morphological characteristics of related *Marasmius lidaensis* species.

Taxa	Pileus size	Pileus color	Basidiospores size	Basidia size	Reference
*M. aurantioferrugineus*	30–70 mm wide	Orange	11.5–15 × 4.5–6.0 μm	26–28 × 8–11 μm	Antonín et al. 2010
*M. brunneospermus*	20–50 mm wide	Yellowish white to light brown	6.0–8.0 × 3.0–4.0 μm	28–32 × 6–7 μm	Antonín et al. 2010
*M. delectans*	10–20 mm wide	Whitish darkened	7–9 × 4 μm	–	Morgan 1905
***M. lidaensis***	**20–40 mm wide**	**Dark orange at center, becoming slightly desaturated yellow with margin**	**5.6–10.2 × 4.5–6.3 μm**	**35–42 × 5–7 μm**	**This study**
*M. maximus*	25–65 mm wide	Ochre-brown at center, otherwise pale brownish to pale ochraceous	7.0–10.3 × 4.5–6.0 μm	35–48 × 7–10 μm	Antonín et al. 2010
*M. nivicola*	5–40 mm wide	White-off, whitish to yellowish white or pale yellow	6.5–8.0 × 3.7–5.0 μm	24–29 × 6–9 μm	Antonín et al. 2010
*M. oreades*	30–55 mm wide	Pale brownish to pale ochraceous	7.5–9.5 × 3.6–5.0 µm	35–48 × 7–10 μm	Razaq et al. 2013
*M. wynneae*	10–50 mm broad	White or gray ochraceous when young, then milky white, gray, or gray violaceous	6.5–8.0 × 3.7–4.5 μm	24 × 7 μm	Antonín et al. 2010

The recognition of these novel taxa not only enriches the known diversity of *Clavaria* and *Marasmius* but also highlights the importance of continued field surveys and integrative taxonomic approaches in understudied areas such as Dali. Our findings not only expand the known fungal diversity of this region but also contribute valuable taxonomic and ecological data for the global fungal inventory. Overall, this study demonstrates that Dali University harbors a surprisingly high diversity of macrofungi and emphasizes the potential for discovering new fungal resources in this region.

**Figure 7.**
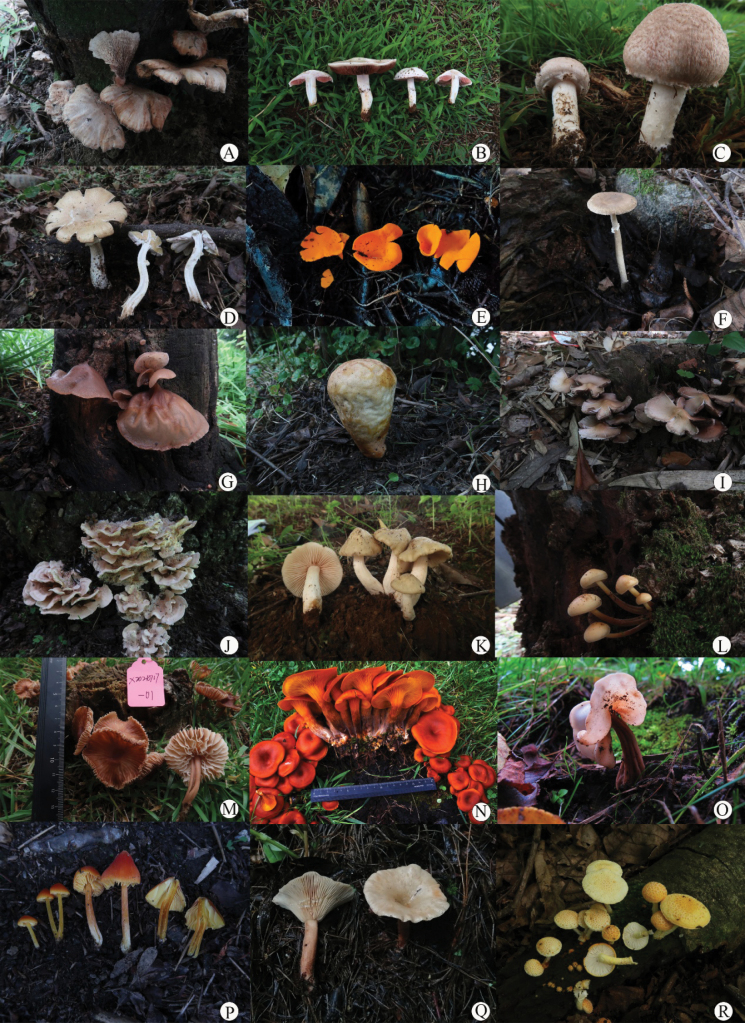
Habitat photos of species collected from Dali University campus. **A**. *Abortiporus biennis*; **B**. *Agaricus campestris*; **C**. *Agaricus cupreobrunneus*; **D**. *Agaricus guizhouensis*; **E**. *Aleuria aurantia*; **F**. *Amanita parvipantherina*; **G**. *Auricularia villosula*; **H**. *Calvatia craniiformis*; **I**. *Candolleomyces candolleanus*; **J**. *Cerrena zonata*; **K**. *Entoloma clypeatum*; **L**. *Flammulina filiformis*; **M**. *Gymnopus dysodes*; **N**. *Omphalotus flagelliformis*; **O**. *Helvella cremeoinvoluta*; **P**. *Hygrocybe coccinea*; **Q**. *Lactarius kesiyae*; **R**. *Cyptotrama asprata*.

**Figure 8.**
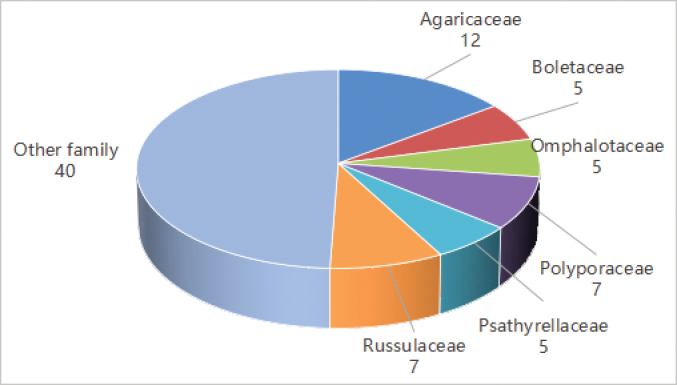
Dominant families of macrofungi in Dali University.

Throughout this survey, 500 macrofungi specimens were collected from the campus and surrounding areas of Dali University. After careful morphological observation combined with molecular analysis, a total of 83 distinct species were recognized (habitat photos of some species see Fig. 7). Among these, the family Agaricaceae proved to be the most species-rich group (see Fig. 8), reflecting its ecological adaptability and dominance in the study area. Following this, representatives of the Polyporaceae and Russulaceae were also frequently encountered, indicating their significant presence and diversity. These findings highlight the broad taxonomic distribution of macrofungi at Dali University and provide important data for regional fungal diversity studies.

Taken together, our findings reveal that the Dali University area harbors a surprisingly high diversity of macrofungi despite its limited spatial extent. This diversity is likely supported by the region’s unique climatic conditions, influenced by the Hengduan Mountain system and the Erhai Lake basin, which generate microclimatic gradients favorable for a wide range of fungal guilds. Additionally, the coexistence of natural, semi-natural, and human-managed habitats around the campus may promote species turnover and maintain fungal diversity through enhanced habitat heterogeneity.

Beyond its scientific implications, this study also holds substantial educational and outreach value. The identification of abundant and visually distinctive macrofungi on campus provides an excellent opportunity for field-based teaching and public science education. Fungal diversity surveys can serve as practical training modules for undergraduate and graduate students, helping them develop skills in taxonomy, ecology, and molecular systematics. Moreover, the documentation and illustration of local fungal species can be incorporated into fungal biodiversity exhibitions, campus nature trails, and digital databases, promoting ecological awareness among students and the broader community. Such initiatives not only enhance biodiversity literacy but also foster a sense of environmental stewardship. The discovery of new species within a university setting vividly demonstrates that scientific discovery can occur in everyday environments, inspiring public curiosity and participation in biodiversity conservation.

In conclusion, this study provides a baseline assessment of macrofungal diversity in the Dali University area and underscores the ecological, taxonomic, and educational significance of campus-based biodiversity research. Continued and expanded surveys across seasons and habitats will be crucial for capturing the full spectrum of fungal diversity and for integrating these findings into long-term monitoring, conservation planning, and science communication efforts in Yunnan and beyond.

## Supplementary Material

XML Treatment for Clavaria lidaensis

XML Treatment for Clavaria minirubella

XML Treatment for Marasmius lidaensis

## Additional information

### Use of AI

No use of AI was reported.

### Author ORCIDs

Song-Ming Tang https://orcid.org/0000-0002-6174-7314

Zheng-Quan Zhang https://orcid.org/0000-0002-2182-3597

Lin Li https://orcid.org/0009-0000-8167-2965

Han-Bing Song https://orcid.org/0000-0002-7440-5444

Zong-Long Luo https://orcid.org/0000-0001-7307-4885

### Data availability

All of the data that support the findings of this study are available in the main text.
